# Advanced Geopolymer-Based Composites for Antimicrobial Application

**DOI:** 10.3390/ma16237414

**Published:** 2023-11-29

**Authors:** Gabriel Furtos, Doina Prodan, Codruta Sarosi, Marioara Moldovan, Michał Łach, Mykola Melnychuk, Kinga Korniejenko

**Affiliations:** 1 “Raluca Ripan” Institute for Research in Chemistry, Babes-Bolyai University, 30 Fantanele Street, 400294 Cluj-Napoca, Romania; codruta.sarosi@gmail.com (C.S.); mmarioara2004@yahoo.com (M.M.); 2 Department of Materials Engineering, Faculty of Materials Engineering and Physics, Cracow University of Technology, 37 Jana Pawła II Av., 31-864 Cracow, Poland; michal.lach@pk.edu.pl; 3 Department of Materials Science, Lutsk National University, 75 Lvivska, 43000 Lutsk, Ukraine; m.melnychuk@lntu.edu.ua

**Keywords:** nanoparticles, geopolymers, graphene oxide, antibacterial, building materials

## Abstract

In most studies about geopolymeric materials used in construction, the antibacterial properties of the building materials are treated as secondary features. Today, antimicrobial properties are a key feature in many building applications. The main objective of this article is to summarize the state-of-the-art in the area of design, development, and applications of nanoparticles as additives to geopolymer composites used in construction to improve their physical mechanical properties and induce a potential antibacterial effect, protecting them against alkali-resistant bacteria. On the basis of the literature and authors’ experience, the most important methods of obtaining especially the porous geopolymers, of nanomaterials used as additives, with potential antibacterial effect but also the potential mechanism of action against bacterial development were presented. The main findings show that using graphene oxide (GO) in geopolymer composites, but also other nanoparticles such as silver (Ag), zinc oxide (ZnO), silica (SiO_2_), titanium dioxide (TiO_2_), copper (Cu) as additives, is an effective way to induce a potential antibacterial effect and to improve the physical and mechanical properties in building materials.

## 1. Introduction

Geopolymers are inorganic polymers obtained by the polycondensation of precursors based on silicon dioxide and aluminum oxide, in a strongly alkaline environment [1]. Although, in the beginning, natural precursors (kaolin, metakaolin, silica fume, and calcined clays) were used to obtain geopolymers, after a while, industrial residues also started being used (fly ash, clay-based slag, etc.). Geopolymers are obtained through the ionic interaction that takes place following the dissolution of Al, Si, and oxygen from the precursors in an alkaline environment by breaking the covalent bond between their atoms. The negatively charged Al (III) attracts alkaline cations (Na^+^, Ca^2+^, K^+^, etc.), followed by the processes of condensation, coagulation, gelation, and polycondensation in three-dimensional networks of silicon and aluminum. The properties of the geopolymer depend on the composition and reactivity of the precursors and, respectively, on the Si/Al ratio of the hydrolyzed species. When reactive Si is predominant, it leads to a larger amount of alkaline aluminosilicate gel, and the Al content contributes to the formation of the network and the chemical structure of the geopolymer [2].

Geopolymers with dense structures have emerged as an alternative to Portland cement, for minimizing or eliminating greenhouse gases. They give building materials high early mechanical strength and good resistance to aggressive atmospheres [3]. Geopolymers with porous structures are materials that can be subjected, especially in a humid environment, to microbial degradation. In dry environments, geopolymers have some antimicrobial protection due to the alkali metal ions in their composition. However, high humidity raises serious problems, contributing to the degradation of geopolymer surfaces or geopolymer concrete by providing a favorable environment for the development of alkali-resistant bacteria, such as those that use sulfur oxidation as a source of energy [4].

The porous geopolymers facilitate antibacterial protection, thanks to the ion exchange of free alkalis in their network, but they can also contribute, depending on the added components, to obtaining a photoactive effect and taming the pH by releasing hydroxyl ions. The properties of porous geopolymer materials can be optimized by adding filler particles, reinforcing components, or those with antibacterial potential, leading to hybrid materials with a synergistic effect by combining the properties of the added components [5].

**Figure 1:**
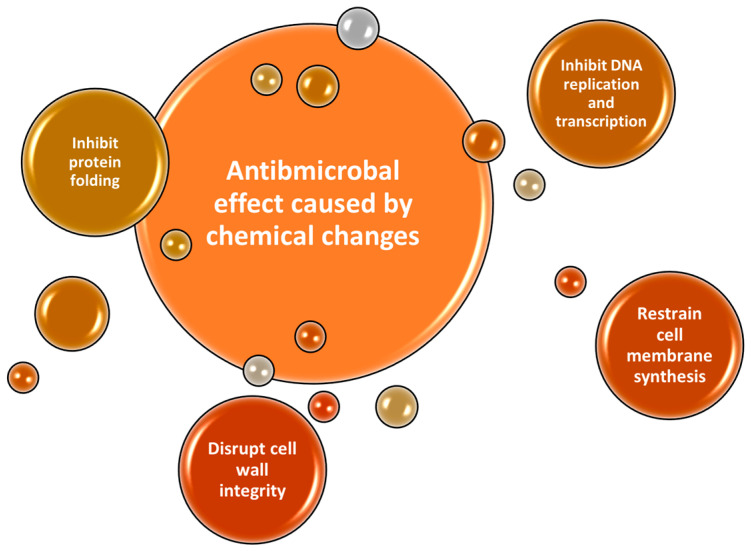
Schematic image of antimicrobial mechanism at surface of materials, caused by ion exchange.

There is a growing interest in the development of geopolymer materials with antimicrobial protection, both to ensure their durability over time and also due to the possibility of their use in environments with high humidity. The addition of antimicrobial additives can be carried out either by directly adding them to the composition (paste) of the materials (mortar, cement, concrete) or by covering their surfaces. The nature of antimicrobial additives can be both inorganic and organic. Inorganic additives (Ag, ZnO, CuO, etc.) can be added in the form of nanoparticles, ensuring antimicrobial protection against a significant number of microorganisms [6]. Figure 1 highlights the different modalities of the nanoparticles from the surface of the materials to fight the bacteria.

Due to their high surface-volume ratio and small dimensions, nanoparticles can be added to the geopolymer matrix, creeping into the pores that form between the larger particles of the majority precursor, providing a filling effect with the help of the alkaline activator [7,8]. Several studies have evaluated the influence of nanomaterials on the properties of geopolymer-based mortars and concretes, both fresh and hardened, in different conditions [9,10]. Most of the reported data are focused on the evaluation of the mechanical properties of building materials based on geopolymers, cured at ambient temperatures [11]. Some authors [12] have investigated the antibacterial effect of a metakaolin-based geopolymer with zinc oxide (ZnO) nanoparticles mixed with sodium hydroxide (NaOH), reporting high mechanical resistance and antibacterial effect. Apart from ZnO, the addition of silver (Ag), titanium oxide (TiO_2_), or copper oxide (CuO_2_) nanoparticles can induce a potential antibacterial and antifungal effect [13,14]. Although these nanoparticles alone can induce an antibacterial effect, several studies have demonstrated that combining them with a geopolymer or with graphene oxide [15] can contribute to synergistic antibacterial activity. The recovery of industrial waste, along with metal oxides or graphene, by including them in the composition of construction materials, can represent a sustainable and cheap alternative way to obtain durable construction materials with improved mechanical properties and potential antibacterial effects. The aims of this review refer to the obtaining methods of nanoparticles, the factors that can influence the obtaining methods, the influence of the added quantities on the properties of the formulated materials, and the potential antibacterial mechanism that nanoparticles can introduce into the materials to which they are added.

## 2. Methods of Obtaining Geopolymers

The denser structure of geopolymers is generally linked to a higher silica content. The abundant silica in the geopolymeric matrix leads to the formation of a denser network, with low porosity and smaller pore size. Therefore, this structure leads to low permeability and an increase in compression resistance. The addition of cellulose or cotton fibers to the composition of geopolymers can also have a good influence on their properties, increasing the mechanical resistance even more [11]. Geopolymers have the ability to stabilize or immobilize certain pollutants or heavy metals and to filter water. Moreover, their adsorption properties help them to stabilize nanoparticles with antimicrobial potential when added to their composition. The geopolymer formulation with a potential antimicrobial effect can prevent the deposition of microorganisms on their surface, preventing microbial degradation [4]. Zhang et al. [5] developed a synthesis of the literature regarding the methods of obtaining other types of geopolymers, with a porous structure. However, porosity and water absorption can lead to a decrease in mechanical resistance and predispose the material to bacterial degradation. Certain inconveniences can be easily solved by adding reinforcing particles or other additives, for example, hydrophobic or antibacterial additives, depending on the scope of application and the expected characteristics. The methods of obtaining porous geopolymers have been classified into two groups: traditional methods and new methods under development. Known traditional methods are: direct foaming and the addition of light or porous filling. One of the most advantageous methods is the use of 3D printing technology due to the possibility of obtaining porous geopolymers with adapted shapes and controlled pore sizes and also due to its flexibility and low costs. Another method of obtaining porous geopolymers is through suspension solidification in order to obtain porous geopolymeric spheres with metakaolin and fly ash for pH buffering applications. The study reports that a higher content of fly ash in geopolymers leads to higher alkaline leaching. The innovative character of these spheres is that they can be handled and collected easily, ensuring gradual and prolonged leaching that no longer requires the addition of hydroxide for continuous pH regulation. This fact could also lead to antibacterial protection including in anaerobic environments [16]. Over time, other methods have also been developed, such as post-grafting, used for the functionalization of porous geopolymers, or the combination of zeolites with porous geopolymers, obtaining superior properties such as an increase in mechanical properties, a large specific surface area, porosity, etc. Other methods are based on the introduction of various fibers (basalt, PVA, polypropylene, cotton, cellulose, etc.) into the composition of porous geopolymers, which can lead to the improvement of mechanical properties [5].

## 3. Nanoparticles Used as Additives in Geopolymers

The use of nanoparticles as additives aims to improve some characteristics of geopolymeric materials. In order to obtain the expected effect, it is very important that these nanoparticles are dispersed uniformly in the mass of the material to avoid agglomerations that could lead to the deterioration of the material’s properties. The most widely used dispersion techniques are ultrasonication, mechanical stirring, or ball milling. Water is a known dispersion medium for the uniform distribution of additives in the material. To increase the homogeneity of the additives by avoiding the formation of agglomerates, surfactants and organic additives can be added to the additives. In order to improve the dispersion of nanoparticles used as additives, it is important that there is a sufficient number of hydrophilic groups in the mixture. These lead to a decrease in the surface tension of water, preventing the formation of agglomerates. From the literature, it appears that certain surfactants used to improve the dispersion can have a negative influence on some additives, namely, on the hydration kinetics of the geopolymer. In this sense, a more appropriate choice is the use of superplasticizers to improve dispersion. It was reported that the 10:1 wt.% ratio of dispersant/nanomaterial led to a reduction in the amount of water used in that study [17]. For this study, we referred to graphene oxide, Ag, Cu, silica, and ZnO, tracking which proportion is more effective. We also find which factors of the method of obtaining can contribute to providing a potential antibacterial effect and to improving the mechanical properties or other properties in the materials in which they are introduced.

### 3.1. Graphene Oxide (GO)

**Figure 2:**
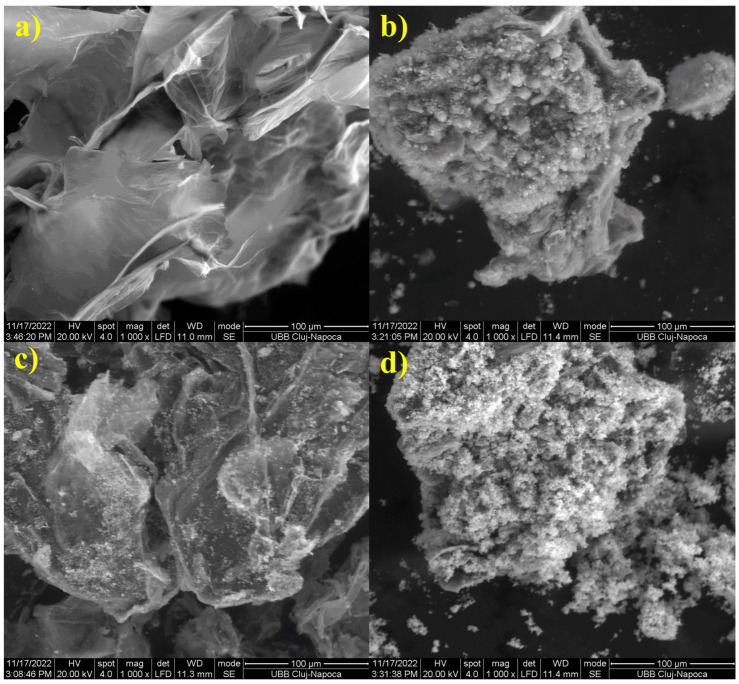
SEM images of the GO: (**a**) GO; (**b**) GO with fly ash; (**c**) GO with silver; (**d**) GO with ZnO at ×1000 magnifications.

Graphene oxide is a versatile material with useful properties for many applications. It is extremely light, having a large specific surface area and good electrical and thermal conductivity. Due to the large specific surface area, GO covers a large contact area with cementing materials, forming a strong network within the cement matrix and ensuring strong adhesion and a dense structure [18]. There are many methods used to obtain GO, which have improved over time. However, each of them has certain advantages and disadvantages. Hummers’ method, e.g., which is based on the use of strong oxidants such as KMnO_4_ and NaNO_3_ to oxidize graphite in an acidic environment, has limited homogeneity. Another method is chemical exfoliation in which graphite layers are separated by oxidation or with the help of solvents. The disadvantage is the toxicity of hydrazine, the most commonly used in this method. The electrochemical exfoliation: uses graphite as a sacrificial electrode, together with exfoliating electrolytes such as: HBr, HCl, HNO_3_, and H_2_SO_4_. The disadvantage of this method is that the obtained graphene has many defects. This method was improved by adding KOH to H_2_SO_4_ (slower oxidation) and combining electrochemical exfoliation with spark plasma sintering (voltage below 10 V), adding surfactants; this has the advantage of being able to obtain graphene on an industrial scale, low price, efficient, environmentally friendly. The arc discharge method has the advantage of large-scale production, graphene has fewer defects, and it is a simple and cheap method [19]. Due to the functional groups with oxygen (hydroxy, epoxy, carboxy) present on the edges and on the surface of the graphene sheets, they can be easily synthesized by chemical oxidation and graphite exfoliation, and they can also be easily functionalized with CuO [20], fly ash, silver, or ZnO [21] Figure 2.

In another study, experimental mortars with potential use in the rehabilitation of heritage buildings were used as additives as 5% GO powder mixtures with ZnO and TiO_2_, and 5% GO powder mixtures with fly ash and Ag. The best antibacterial effect was reported for the mortar with the GO powder mixture with ZnO and TiO_2_ as additives. This result is due to the fact that the concentration of Ag in the additive was much lower than that of Zn and Ti. However, the result was very encouraging, with the values of the diameter of the bacterial inhibition zone being very close in both cases [22]. The photonic properties of GO can improve the photocatalytic properties of other materials [23]. Long et al. [24] added different percentages of GO (0.05 wt.%, 0.1 wt.%, and 0.2 wt.%) to cement using a water-cement ratio (W/C) of 0.66. After 28 days, the measured flexural strength of the mortar samples was higher with 16 wt.%, 27 wt.%, and 41 wt.% compared to the mortar without GO. Moreover, the compressive strength increased by approximately 7%, 10%, and 10%, respectively. In another study, Wang et al. [25] reported that a cement paste with a W/C ratio of 0.40 in which different wt.% percentages of GO were added (0.02 wt.%, 0.04 wt.%, 0.06 wt.%, and 0.08 wt.%) increased the values of the compressive and bending strengths after 1, 3, 7, and 28 days. By adding 0.02 wt.% GO, the strengths were only slightly higher compared to the sample without GO. By adding 0.04 wt.% GO, after 28 days, the compressive and flexural strengths increased by 20% and 23%, and for 0.08 wt.% GO, increases of 16% and 27 wt.% were recorded.

### 3.2. Silver (Ag)

The antibacterial effect of Ag nanoparticles has been known since ancient times. This effect is due to silver ions (Ag+), which once released, prevent the formation of an enzyme like adenosine triphosphate (ATP) through energy storage, but also through the replication of bacterial DNA, thus inducing bacterial death [14]. There are several physical, chemical, or biological methods to obtain Ag nanoparticles. By ball milling, laser ablation, or sputtering [26], large quantities of nanoparticles can be obtained, but depending on the chosen technique, the obtained nanoparticles can show a higher purity. The disadvantages of this method include high energy consumption, expensive equipment and high pressure and temperature [27]. Electrochemical reduction, the sol-gel method, or chemical reduction, besides being considered cheap methods, require only a metal precursor, a reducing agent, and a stabilizing agent. They are simple to perform, but also controllable, leading to the obtaining of spherical nanoparticles [28]. The disadvantage of these chemical methods would be the pollution generated from the use of toxic reagents or solvents [29]. Most often, nanoparticles are obtained by the sol-gel method. In the first stage, the goal is to obtain a stable metal precursor through hydrolysis. The condensation leads to obtaining a metal hydroxide network. These stages are influenced by a series of parameters like type of solvent, pH, temperature, catalyst, additives, etc. Finally, very fine particles with the desired crystallinity are obtained by drying and heating [30]. The syntheses that use natural extracts as reagents [31] to obtain Ag nanoparticles ensure high yield and stability [32] and have low costs due to the variety of natural products.

Geopolymer nanocomposites can also be used for water treatment. The addition of 0.05 wt.% silver nanoparticles and hydrogen peroxide foam to a composite based on aluminosilicate (bentonite) for water filters against bacteria had a low efficiency against coliphages. However, the efficiency increased against *E. coli* and *enterococci* bacteria [4]. Some characteristics of porous geopolymers with Ag or Cu additives (0.05 wt.%) were compared and introduced, using several methods, into geopolymers, with the aim of obtaining water filters with potential disinfectant or catalytic properties. The compressive strength values for the samples obtained by three different methods were: 16 MPa (by 3D printing), 1 MPa (by direct foam), and 10 MPa (granulation). The amount, state of oxidation, and stability of the additives were influenced by both the filter preparation method and the additive impregnation method. The largest amount of Ag was obtained by the granulation method in which Ag was introduced by immersing the filter in a colloidal Ag solution, compared to adding it to the fresh paste [33].

### 3.3. Zinc Oxide

Synthesis of ZnO nanoparticles by the sol-gel technique has the advantage of low cost and diverse applicability, improving the properties of many polymers [34]. Like Ag, Zn nanoparticles can also be obtained by biological methods which are more environmentally friendly. Bhuyan et al. [35] obtained spherical nanoparticles of ZnO, using *Azadirachta indica* (Neem) leaf extract, which showed good antimicrobial activity against *Staphylococcus aureus*, *Streptococcus pyogenes*, and *Escherichia coli*. In general, the production of nanoparticles is observed by the changing color of the reaction mixture [36] and can be confirmed by Fourier Transform Infrared Spectroscopy (FTIR), identifying the biomolecules involved in the reaction [37]. In their study, Singh et al. [38] evaluated the antimicrobial effect of cement with additions of 0.5, 10, and 15 wt.% ZnO against *Escherichia coli*, *Bacillus subtilis*, and *Aspergillus niger*. It was observed that the antibacterial and antifungal effects of cement increased with the increase in ZnO concentration and were enhanced by sunlight. Wang et al. [39] investigated the antimicrobial effect of high-performance concrete with ZnO addition and found that the antibacterial effect was 100% against *Escherichia coli* and 54.61% and 99.12%, respectively, against *Staphylococcus aureus*. In the literature, there are reports about the addition of supplements with antimicrobial properties to building materials (concrete, mortar, bricks, etc.), which do not significantly affect the essential properties of the materials [40].

### 3.4. Silica Nanoparticles and Silica Fume

Silica nanoparticles can be obtained by dry or wet methods. In the dry method category, the gas phase method and the arc method can be mentioned. Some wet methods include precipitation, sol-gel, microemulsion, and high-gravity reaction [41]. Stefanidou and Karazou [42] tested bricks treated with linseed oil, silane/siloxane, and alcosiloxane modified with 1–1.5 wt.% silica nanoparticles and demonstrated that the alcosiloxane and silica nanoparticles protect the bricks, providing a high resistance to water absorption and an increase in their durability. Zhang et al. [43] studied the influence of different nanoparticles introduced into the mortar and investigated the resistance and hydration mechanism for the cement mortar. It was reported that the added nanoparticles, formed bonds with the matrix, leading to an increase in the strength of the samples and a decrease in the initial and final setting time of the matrix. Comparing nano-silica with silica fume, a study reported that only 75 wt.% of silica fume was consumed after 90 days [43], while nano-silica, due to its high specific surface, can play a nucleation role in the process of cement hydration, accelerating this process and improving early strength [41]. By adding nano-silica, a denser microstructure can be obtained, due to the penetration of nanoparticles into the pores of the material making it possible to effectively control the release of calcium hydroxide, which can influence the corrosion of concrete, thus increasing its strength and durability [43,44]. Wang et al. [45] believed that the graphene nanosheets can accelerate the hydration process with the formation of a compact microstructure.

### 3.5. Copper

As in the case of Ag or Zn, Cu nanoparticles can be obtained by physical, chemical, or biological methods. The major problem is that the Cu particles tend to oxidize in the air, resulting in an agglomeration of the particles. To avoid this shortcoming, the Cu nanoparticles are obtained in an inert gas atmosphere [46]. Moreover, to avoid oxidation, protective polymers or surfactants can be used [47].

Using chemical methods, the size and shape of the particles can be controlled by adjusting the pH and the ratio between the electrolyte and the surfactant with the help of a protective agent, varying the type of solvent, etc. However, obtaining large quantities is not possible. An alternative to this method is needed, which should require cheaper reagents and an easier reaction condition. Therefore, biological methods can be considered a safer alternative, based on bio-organisms or plants requiring ecological reagents [48]. They are considered to be the most convenient methods because, in addition to plant extracts, they use bacteria, fungi and algae, and phytochemicals from plants, which act both as reducing agents and as stabilizers [46]. In the case of mortar mixed with copper powder, the compressive and flexural strengths were higher than those of the reference mortar [48]. Table 1 shows the most used methods of obtaining nanoparticles used as additives in geopolymers and the effect they have on some properties of the geopolymers in which they are introduced.

## 4. The Antibacterial Mechanism of Nanoparticles Used as Additives in Building Materials

**Figure 3:**
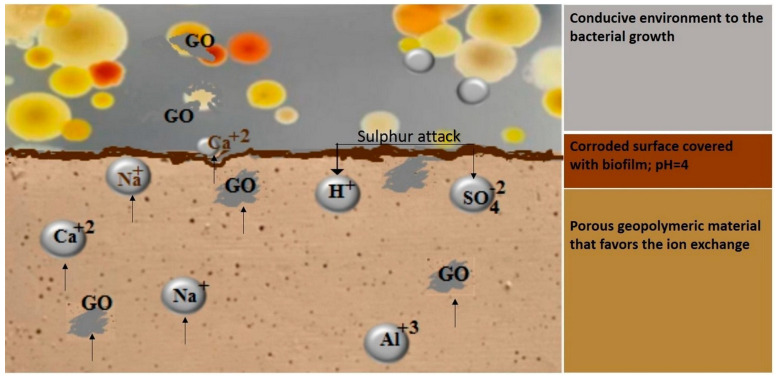
Schematic representation of the surface of the geopolymeric material after acid attack followed by microbial action and the potential antibacterial mechanism of the graphene oxide nanosheets.

The addition of Ag, Cu, TiO_2_, ZnO, or GO nanoparticles in the geopolymer matrix can lead to an increase in resistance to compression, dehumidification, and a potential antibacterial effect. Bacteria develop on the surface of any material if they have favorable conditions such as a suitable temperature, a source of nutrients that can be absorbed and decomposed by enzymes to provide the energy necessary for bacterial growth, a suitable pH, and a gaseous environment. At a pH value between 12 and 13, bacteria will not grow on the surface of the freshly prepared material. However, there are environments, such as sewage, where, after a while, an increased concentration of H_2_S accumulates. In the case of sewer pipes, it was found that sulfate ions deposited on the material in an anaerobic environment. These are reduced by sulfate-reducing bacteria, leading to the formation of H_2_S. In contact with the pipe walls, H_2_S is oxidized to H_2_SO_4_ by sulfur-oxidizing bacteria in the air. Figure 3 presents a case of a porous geopolymer, with graphene oxide as an additive used for sewage, which, in a closed environment, suffered an acid attack. Especially in the case of sewers where there is water and the bacteria have a large amount of nutrients, concrete corrosion is more serious. The nutrients of sulfur-oxidizing bacteria are provided by heterotrophic fungi that break down organic substances with sulfur even in a large pH range. Through the action of microorganisms, other acids can be produced, such as HNO_3_, acetic acid, or oxalic acid, which can also lead to concrete corrosion. When the pH inside drops below 9, microorganisms multiply. These microorganisms will provide acid, and the pH can drop even more [49]. Around a pH of 4, there is a massive bacterial proliferation, leading to the worsening of concrete corrosion. The advantage of geopolymer concrete compared to plain concrete is that the microorganisms have a larger volume than the pore size, the material being more compact, and the acid metabolism takes place only on the surface of the material, making the corrosion process more difficult [50]. Data from the literature report that physical contact can cause the destruction of bacteria, through the ion exchange mechanism. The divalent cations in the outer wall of the cell membrane can be dislocated by the cations on the surface of the material loaded with antimicrobial additives, leading to cell death. The authors are of the opinion that the sharp tips that come out of the surface of a material (for example, graphene oxide has sharp edges) can pierce the cell wall, causing the leakage of the cytoplasm and finally cell death [51]. The mechanism of inducing the antibacterial effect depends on the type, shape, and size of the added nanoparticles and is explained below.

### 4.1. Graphene Oxide

It is supposed that one of the mechanisms for the antibacterial effect is of a mechanical nature. Due to the shapes of the graphene nanosheets, the “sharp” edges, could section the cell membrane, leading to the leakage of the intracellular matrix, and finally, to its death. Another explanation could be that larger sheets of GO can wrap the bacteria, preventing their proliferation. A study proposes, that the antibacterial activity is not due to reactive oxygen species (ROS) but to the transfer of electrons between the bacterial membrane and the graphene surface [52]. It is believed that oxidative stress does not depend on ROS because graphene acts as an electron acceptor and its surface is important in inducing the antibacterial effect and not its edges. Therefore, GO does not kill the bacteria but inactivates them [53]. However, there could be some doubts: the possibility that the GO sheets are too thick and their edges could not penetrate the bacterial membrane or if the rigidity of the nanosheets does not allow them to wrap the bacteria. For this, an experiment was carried out in which the Langmuir–Blodgett technique was used [54]. The GO sheets, including the edges, were immobilized on a substrate, and then the antimicrobial test was performed. It was concluded that the antimicrobial mechanism of GO depended on the surface of the GO sheets, which can lead to bacterial inactivation in different ways, and does not depend at all on their edges [55].

**Figure 4:**
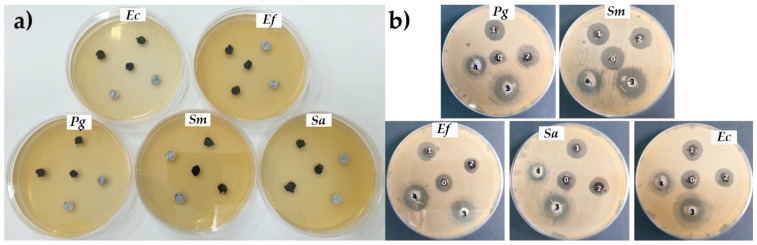
Disk diffusion assay on *Porphyromonas gingivalis* (Pg), *Streptococcus mutans* (Sm), *Staphylococcus aureus* (Sa) and *Escherichia coli* (Ec) for the GO powders [21].

There are studies that have demonstrated the antibacterial effect of graphene oxide [21]. Figure 4 shows the antibacterial effect of different graphene powders: GO, GO-Ag, GO-Zn, GO-APTES, GO-Ag-APTES, and GO-Zn-APTES against five bacterial strains (*Streptococcus mutans* ATCC 25175, *Porphyromonas gingivalis* ATCC 33277, *Enterococcus faecalis* ATCC 29212, *Escherichia coli* ATCC 25922, and *Staphylococcus aureus* ATCC 25923) by measuring the zone of inhibition after 18–24 h incubation at 37 °C.

### 4.2. Silver

The opinion of some authors is that Ag can induce an antibacterial effect in three ways. It is supposed that the Ag nanoparticles can penetrate the outer membrane and then surround the inner membrane, increasing its permeability favoring the leakage of the cell content and then death [56]. On the other hand, the Ag nanoparticles can cause damage due to interactions with sulfur proteins, located in the cell wall, leading to the rupture of the cell wall. Another possibility of inducing the antibacterial effect is that the Ag nanoparticles can penetrate and cross the cell membrane, entering the cell, where they could interact with the DNA and the proteins, disturbing the structure and functions of the cell. On the other hand, Ag nanoparticles can interact with enzymes through thiol groups, inducing reactive oxygen species, leading to the impairment of intracellular functions, and inducing apoptosis. The third way can appear at the same time as the other two and refers to the release of ions from Ag nanoparticles. Ag ions can interact with cellular components affecting cellular metabolism and genetic material [57]. Silver nanoparticles are also used by combining with carbon fibers against bacteria and other harmful microorganisms [58]. This composition is sometimes enriched with organic compounds, such as antibiotics (gentamicin) to improve antimicrobial properties. One of the popular antibacterial additives is also silver salts, especially silver nitrate [58]. One study [33] reported adding a composite foam prepared with 0.05% silver nanoparticles as an additive, to geopolymers based on bentonite for the purpose of water disinfection. In time, the effect against *coliphages* decreased, but against *E. coli* and *enterococci* bacteria, it was significant.

### 4.3. Zinc Oxide (ZnO)

For zinc, the most accepted antibacterial theory is based on the generating of reactive oxygen species (ROS). Active oxygen occurs from reducing oxygen O^2+^ e^−^ → *O^2−^ and the most common ROS are peroxides, hydroxyl radicals, and singlet oxygen. Because OH- cannot penetrate the cell membrane; is placed on its walls, disrupting cellular functions by destroying the membrane. Hydrogen peroxide (H_2_O_2_) can penetrate the cell membrane, causing its destruction and the destruction of DNA, which has a bactericidal role [59]. By releasing it from ZnO, Zn^2+^ can destroy the cell membrane leading to the inhibition of cell proliferation due to the denaturation of proteins and the disruption of the cellular energy mechanism by disturbing the electron transport. Although there are numerous reports in the literature attributing the potential antibacterial effect of Zn after catalysis, it was demonstrated that a strong antibacterial effect was also obtained in dark conditions [60]. The opinion of some researchers is that the antibacterial effect of Zn does not increase with the rise in Zn^2+^ concentration. It can be assumed that the destruction of bacteria can take place due to electrostatic attraction between positively charged Zn^2+^ and the surface of the cell membrane, which is negatively charged, by disturbing the charge balance of the membrane surface [61].

### 4.4. Silica (SiO_2_)

In order to find an alternative for Portland cement in concrete, a geopolymer based on fly ash was proposed, improved by the addition of ZnO nanopowder (rods) covered with spherical silica nanoparticles in order to protect against corrosion, to improve mechanical resistance, and to fight against: *E. coli*, *S. aureus* and *A. niger*. The tests showed that the geopolymer with Zn-SiO_2_ nanohybrid powder has a bactericidal action rather than a bacteriostatic one. The cells that were treated with the Zn-SiO_2_ nanohybrid generated an approximately four times higher ROS level compared to the reference for the microbial strains. Although the effect against microbial species was mainly due to Zn, it has been proven that the combination of the proposed nanohybrid is successful both for protection against chemical corrosion and against biological corrosion. High mechanical strengths were also recorded [62].

### 4.5. Copper (CuO)

Like Ag, the Cu ions from nanoparticles can interact with the DNA and proteins, disrupting the cellular biochemical structure and process. A study [63] proposed the obtaining of cement mortars for the treatment of waste water and for the inhibition of bacterial growth. They obtained Cu-Ti alloys in atomic ratios: 35:65, 50:50 and 65:35 by ball grinding. The authors of this study investigated the influence of different concentrations of amorphous Cu-Ti alloy (from 0.3 to 0.9 wt.%) added to mortars, together with 0.5% superplasticizer. The Cu-Ti alloy (35:65) has the finest particles, leading to an increase in the mechanical properties of the mortar. The mortar with 0.9 wt.% Cu-Ti alloy (50:50) recorded the best antibacterial effect. Another study [64] showed that cell filamentation is caused by membrane depolarization, under the influence of Cu nanoparticles, more precisely under the influence of Cu^2+^ ions, resulting from the oxidation of the metallic Cu atoms of the nanoparticles. The destruction of cells is caused by the production of ROS under the action of nanoparticles, leading to DNA degradation, together with lipid peroxidation and protein oxidation. Table 2, there are shortly summarizes the most popular theories about the antimicrobial effects of selected nanoparticles.

## 5. Trends in Geopolymer Nanocomposites Applications as Building Materials

The use of nanomaterials in the building industry become more and more popular [65,66] but the most obvious application is the antimicrobial application in the infrastructure dedicated to health care [27,32]. By combining the carbon fibers with silver nanoparticles or silver nitrate additives [67] a new area of application in the design of health infrastructure with aseptic properties was opened. Due to the absorption properties of porous geopolymers, they can offer real potential in medical applications for the adsorption of specific biomolecules. In recent years, the human organism has become increasingly resistant to the action of antibiotics. An alternative method to avoid dependence on the consumption of antibiotics would be to obtain porous geopolymers, with controlled porosity, capable of removing the pathogenic material and having a preventive role to avoid infections in hospitals. The area of applicability of these materials could be extensive, to avoid potential infections in poultry farms, in aquaculture, in agriculture, and even for the delivery of medicines [68]. The other application is connected with the functionalization of materials to provide antimicrobial properties for vulnerable materials to microbially-induced degradation in specific environments allowing for protection of the materials against bio-corrosion or control the material colonization [4,22]. Zhang et al. [69] obtained hybrid materials, based on geopolymers with metakaolin, functionalized with (3-aminopropyl) triethoxysilane (APTES) for surface protection and for improving the workability of mortars. It was found that APTES slow down the geopolymerization reaction and in their opinion there is a slow decomposition of the geopolymer network that recombines with the silane forming a more homogeneous network and much more resistant to compression. The functionalization of porous geopolymers with hydrophobic agents, such as polydimethylsiloxane, for the protection of their surfaces against moisture, also represents another direction of their use [4]. Alkali-activated foams based on coal gangue used as porous pH regulators, having high porosity and high resistance, also have a great perspective. In addition to the fast method of obtaining and the possibility of recycling the waste, it also offers anti-corrosion protection. They could also have antibacterial potential due to the gradual and prolonged alkali leaching [70].

## 6. Conclusions

The use of GO in building materials implies the achievement of cleaner production and the support of “green technologies”. It can be considered as the best option to protect the environment and reuse current resources, as abundantly available precursors. This study is a review of the opportunity of using GO and graphene oxide in building materials, but also other nanoparticles such as Ag, ZnO, SiO_2_, and Cu as additives, in order to induce a potential antibacterial effect. The methods of obtaining these nanoparticles are briefly presented, as the influence of different quantities of nanoparticles on some material properties, as well as examples of characterization techniques that can highlight the presence of these additives in building materials. Also, the mechanism of achieving antibacterial properties is briefly presented and correlated with the used nanoparticles. These nanoparticles can be included in the building materials, as additives, alone or together with GO and/or geopolymers. Due to the small size of nanoparticles, there is a risk of their release from the network of the geopolymer material into the environment, which should be considered and monitored for each material before application.

## Figures and Tables

**Table 1:** The most used methods of obtaining nanoparticles; nanoparticles’ effect on geopolymer properties.

NPs	Obtaining Methods	Sources	Effects on Geopolymers	Sources
GO	Hummers’ method Chemical exfoliation Electrochemical Exfoliation Arc discharge	[19]	Strong adhesion and a dense structure Improve the photocatalytic properties Induce an antibacterial effect Increasing flexural and compression resistance	[18] [23] [22] [24,25]
Ag	Ball milling Electrochemical reduction Sol-gel method, Chemical reduction	[26,28]	Antibacterial properties against *E. coli* and *enterococci bacteria* The mechanical strengths depend on the NP addition mode	[4] [33]
ZnO	Sol-gel technique Biological method	[34,35]	Antimicrobial effect against *Escherichia coli*, *Bacillus subtilis*, *Aspergillus niger* and *Staphylococcus aureus*	[35,38,39]
Silica NPs and silica fume	Gas phase method Arc method Precipitation Sol–gel method Microemulsion, High-gravity reaction	[41]	Resistance to the water absorption of the bricks Decrease in the initial and final setting time of the matrix Preventing corrosion and increasing strength and durability of concrete	[42] [43] [43,44]
Cu	Physical methods Chemical methods Biological methods	[46,48]	Increasing compression and flexural resistances	[48]

**Table 2:** The main antibacterial mechanism for selected nanoparticles.

Nanoparticle	Main Antibacterial Mechanism	Sources
GO	Larger sheets of GO can wrap the bacteria; “sharp” edges, could destroy the cell membrane.	[52,53,55]
Ag	Distorting the cellular membrane; Interaction with the DNA and proteins, especially sulfur proteins.	[56,57]
ZnO	Generating of reactive oxygen species.	[59,60,61]
SiO_2_	Zn-SiO_2_ nanohybrid generating reactive oxygen species	[62]
Cu	Interaction with the DNA and proteins.	[63,64]

## Data Availability

The data that support the findings of this study are contained within the article.
